# Supplementary material for: Preventive behaviors of COVID-19 during the COVID-19 pandemic among community-dwelling older adults in Thailand
Source: PeerJ. 2025 May 13;13:e19412. doi: 10.7717/peerj.19412 (PMC12083464; doi:10.7717/peerj.19412)
Supplement: Supplemental Information 2 [file peerj-13-19412-s002.pdf]

เลขที่แบบสัมภาษณ์...........

แบบสัมภาษณ์

โครงการวิจัยเรื่อง พฤติกรรมป้องกันตนเองระหว่างการระบาดของ COVID-19

ของผู้สูงอายุที่อาศัยอยู่ในชุมชน ในประเทศไทย

คำชี้แจง แบบสัมภาษณ์ชุดนี้แบ่งเป็น 4 ส่วนประกอบด้วย

ส่วนที่ 1 ข้อมูลส่วนบุคคล

ส่วนที่ 2 แบบสัมภาษณ์พฤติกรรมการป้องกันโรคติดเชื้อไวรัสโคโรนา 2019

ส่วนที่ 3 แบบสัมภาษณ์ความรู้เรื่องโรคติดเชื้อไวรัสโคโรนา 2019

ส่วนที่ 4 แบบสัมภาษณ์การรับรู้ความเชื้อด้านสุขภาพเกี่ยวกับโรคติดเชื้อไวรัสโคโรนา 2019

ผู้สัมภาษณ์ดำเนินการสัมภาษณ์และบันทึกข้อมูลจากการสัมภาษณ์ หรือทำเครื่องหมาย √ ลงในช่อง ( ) หน้าข้อความ และเติมข้อความลงในช่องว่างให้ครบทุกข้อ ข้อมูลจากการสัมภาษณ์ในครั้งนี้ ถือเป็นความลับ และไม่มีผลเสียหายต่อผู้ให้สัมภาษณ์แต่อย่างใด ซึ่งผู้วิจัยจะนำข้อมูลที่ได้ไปวิเคราะห์และนำเสนอผลการวิจัยในภาพรวมต่อไป ผู้วิจัยจึงขอความร่วมมือจากผู้ให้สัมภาษณ์ โปรดให้คำตอบตามความเป็นจริงจะเป็นพระคุณยิ่ง ทั้งนี้เพื่อประโยชน์ในการศึกษาวิจัยครั้งนี้ ท้ายนี้ผู้วิจัยขอขอบคุณในความร่วมมือในการตอบแบบสัมภาษณ์มา ณ โอกาสนี้

BUU-IRB Approved
21 May 2021

ส่วนที่ 1 ข้อมูลส่วนบุคคล

คำชี้แจง ผู้สัมภาษณ์ทำการสัมภาษณ์ผู้สูงอายุและทำเครื่องหมาย √ ลงในช่อง ( ) ที่ตรงกับคำตอบของผู้สูงอายุมากที่สุดหรือเติมข้อความลงในช่องว่าง.........ให้สมบูรณ์

1. เพศ ( ) 1 ชาย ( ) 2 หญิง

2. ปัจจุบันท่านมีอายุ.......................ปี

3. สถานภาพสมรสในปัจจุบันของท่าน ( ) 1 สมรส ( ) 2 โสด ( ) 3 หม้าย ( ) 4 หย่า/แยก

4. ท่านนับถือศาสนา ( ) 1 พุทธ ( ) 2 อิสลาม ( ) 3 คริสต์ ( ) 4 อื่น ๆ (ระบุ) ....

5. ปัจจุบันท่านประกอบอาชีพ

( ) 1 ไม่ได้ทำงาน ( ) 2 แม่บ้าน ( ) 3 เกษตรกรรม ( ) 4 รับจ้าง

( ) 5 ธุรกิจส่วนตัว/ค้าขาย ( ) 6 รับราชการบำนาญ ( ) 7 อื่น ๆ (ระบุ) ....

6. ปัจจุบันท่านมีรายได้เฉลี่ย..................................บาทต่อเดือน

7. ระดับการศึกษาของท่าน

( ) 1 ไม่ได้เรียนหนังสือ ( ) 2 ประถมศึกษาปีที่ 4 ( ) 3 ประถมศึกษาปีที่ 6

( ) 4 มัธยมศึกษาตอนต้น ( ) 5 มัธยมศึกษาตอนปลาย/ปวช ( ) 6 อนุปริญญา/ปวส.

( ) 7 ปริญญาตรี ( ) 8 สูงกว่าปริญญาตรี ( ) 9 อื่น ๆ ระบุ......................

8. ท่านคิดว่าสุขภาพของท่านในปัจจุบันนี้เป็นอย่างไร

( ) 1 ท่านมีสุขภาพดีมาก ( ) 2 ท่านมีสุขภาพดี

( ) 3 ท่านมีสุขภาพดีพอใช้ ( ) 4.ท่านมีสุขภาพไม่ดี

9. ปัจจุบันท่านมีโรคประจำตัวหรือไม่ ถ้าท่านมีโรคประจำตัวท่านมีโรคประจำตัวดังต่อไปนี้หรือไม่

( ) 0 ไม่มี ( ) 1 มี (ตอบได้มากกว่า 1 ข้อ)

( ) 1 โรคความดันโลหิตสูง ( ) 2 โรคเบาหวาน ( ) 3 โรคหัวใจและหลอดเลือด

( ) 4 โรคปอดอุดกั้นเรื้อรัง ( ) 5. ภาวะไขมันในเลือดสูง ( ) 6 เก๊าต์

( ) 7.ภูมแพ้ ( ) 8 ปวดกระดูก/ข้อ ( ) 9 โรคไตวายเรื้อรัง

( ) 10 อื่นๆ ระบุ.......................................

BUU-IRB Approved
21 May 2021

ส่วนที่ 2 แบบสัมภาษณ์พฤติกรรมการป้องกันโรคติดเชื้อไวรัสโคโรนา 2019

คำชี้แจง ผู้สัมภาษณ์ทำการสัมภาษณ์ผู้สูงอายุตามข้อคำถามเกี่ยวกับพฤติกรรมการป้องกันโรคติดเชื้อไวรัสโคโรนา 2019 แล้วให้ทำเครื่องหมาย ✔ ในข้อที่ตรงกับการปฏิบัติที่ผู้สูงอายุระบุมาดังนี้

ปฏิบัติเป็นประจำ หมายถึง ผู้สูงอายุปฏิบัติกิจกรรมนั้นสม่ำเสมอทุกครั้ง ให้ 3 คะแนน
ปฏิบัติบ่อยครั้ง หมายถึง ผู้สูงอายุปฏิบัติกิจกรรมนั้นได้เป็นส่วนใหญ่ ให้ 2 คะแนน
ปฏิบัติบางครั้ง หมายถึง ผู้สูงอายุปฏิบัติกิจกรรมนั้นได้บ้างบางครั้ง ให้ 1 คะแนน
ไม่เคยปฏิบัติ หมายถึง ผู้สูงอายุไม่เคยปฏิบัติกิจกรรมนั้น ๆ ให้ 0 คะแนน

พฤติกรรมการป้องกันโรคติดเชื้อไวรัสโคโรนา 2019	ปฏิบัติเป็น [illegible]	ปฏิบัติบ่อย ครั้ง	ปฏิบัติบ้างครั้ง	ไม่เคยปฏิบัติ	สำหรับผู้วิจัย
	3	2	1	0	
1. ท่านสวมใส่หน้ากากอนามัย หรือหน้ากากผ้าป้องกันทุกครั้งเมื่อออกไปทำธุระ นอกบ้าน					BH1 ( )
2. ท่านล้างมือด้วยสบู่ หรือเจลแอลกอฮอล์ ทุกครั้งเมื่อสัมผัสสิ่งของร่วมกับผู้อื่น หรือก่อนรับประทานอาหาร หรือ หลังเข้าห้อมส้วม					BH2 ( )
3. ท่านหลีกเลี่ยงการใช้มือสัมผัส ใบหน้า ตา ปาก จมูก					BH3 ( )
4. ในช่วงที่มีการระบาดของโรค ท่านหลีกเลี่ยงการไปในสถานที่ที่มีคนแออัด เช่น ไปซื้อของตลาด ไปทำบุญที่วัด ไปห้างสรรพสินค้า หรือการเข้าร่วมกิจกรรมต่าง ๆ ซองชุมชน หรือของชมรมผู้สูงอายุ					BH4 ( )
5. ท่านออกกำลังกายอย่างน้อย 3 วันต่อสัปดาห์เพื่อให้มีสุขภาพแข็งแรง และ ป้องกันการติดเชื้อไวรัสโคโรนา 2019					BH5 ( )
6. ท่านรับประทานอาหารครบ 5 หมู่ อย่างน้อย 3 มื้อ ต่อวัน เพื่อให้มีสุขภาพแข็งแรงและป้องกันการติดเชื้อไวรัสโคโรนา 2019					BH6 ( )
7. ท่านรับประทานยาสมุนไพรพื้นบ้านทั้งสมุนไพรไทย หรือสมุนไพรจีน เพื่อป้องกันการติดเชื้อไวรัสโคโรนา 2019					BH7 ( )
8. ท่านรับประทานวิตามิน หรืออาหารเสริม หรือ ยาฆ่าเชื้อ เพื่อป้องกันการติดเชื้อไวรัสโคโรนา 2019					BH8 ( )

BUU-IRB Approved
21 May 2021

ส่วนที่ 3 แบบสัมภาษณ์ความรู้เรื่องโรคติดเชื้อไวรัสโคโรนา 2019

คำชี้แจง ผู้สัมภาษณ์ทำการสัมภาษณ์ผู้สูงอายุตามข้อคำถามเกี่ยวกับ**ความรู้เรื่องโรคติดเชื้อไวรัสโคโรนา 2019** แล้วให้ทำเครื่องหมาย ✔ ในข้อที่ตรงกับความคิดเห็นของผู้สูงอายุระบุมาดังนี้

ใช่ หมายถึง ข้อความนั้นตรงกับความคิดเห็นของผู้สูงอายุ
ไม่ใช่ หมายถึง ข้อความนั้นไม่ตรงกับความคิดเห็นของผู้สูงอายุ
ไม่แน่ใจ หมายถึง ผู้สูงอายุไม่รู้ หรือแน่ใจในคำตอบในข้อคำถามนั้น

**รายการ**	ใช่	ไม่ใช่	ไม่แน่ใจ	ผู้วิจัย
1. การสวมหน้ากากอนามัยหรือหน้ากากผ้าเป็นประจำ และการล้างมือบ่อย ๆ สามารถช่วยป้องกันการติดเชื้อไวรัสโคโรนา 2019 ได้				Kn1 ( )
2. เมื่อผู้ที่ติดเชื้อไวรัสโคโรนา 2019 ไอ หรือจามในที่สาธารณะจะทำให้คนรอบข้างติดเชื้อไวรัสโคโรนา 2019 ได้ง่าย				Kn2 ( )
3. การมีภูมิคุ้มกันไวรัสโคโรนา 2019ในร่างกาย สามารถป้องกันการติดเชื้อไวรัสโคโรนา 2019ได้				Kn3 ( )
4. การหลีกเลี่ยงไปในสถานที่ที่แออัด หรือสถานที่ ที่คนไปชุมนุมกันมาก ๆ จะช่วยป้องกันการติดเชื้อ ไวรัสโคโรนา 2019ได้				Kn4 ( )
5. ผู้ที่ติดเชื้อไวรัสโคโรนา 2019 จะมีอาการไข้ อ่อนเพลีย ไอแห้ง ๆ และปวดเมื่อยกล้ามเนื้อ				Kn5 ( )
6. ผู้ที่ติดเชื้อไวรัสโคโรนา 2019 จะมีอาการไอ จาม คัดจมูก มีน้ำมูกไหลน้อยกว่าผู้ที่เป็นไข้หวัด				Kn6 ( )
7. ปัจจุบันยังไม่มีวิธีการรักษาที่โรคติดเชื้อไวรัสโคโรนา 2019 ที่มีประสิทธิภาพ ยกเว้นการป้องกันตนเองจากการสัมผัสเชื้อโรค				Kn7 ( )
8. ผู้สูงอายุที่ป่วยเป็นโรคเรื้อรังและมีภาวะอ้วน เมื่อติดเชื้อไวรัสโคโรนา 2019 แล้วจะมีอาการรุนแรงกว่าผู้อื่น				Kn8 ( )
9. เชื้อไวรัสโคโรนา 2019 แพร่กระจายเชื้อทางละอองฝอยของผู้ติดเชื้อที่ ไอ หรือจาม ออกมา				Kn9 ( )
10. ผู้ที่ติดเชื้อไวรัสโคโรนา 2019 ที่ไม่มีอาการไข้ ไอ เจ็บคอ ไม่สามารถแพร่เชื้อไปยังผู้อื่นได้				Kn10 ( )
11. ผู้ที่สัมผัสกับผู้ติดเชื้อไวรัสโคโรนา 2019 ควรต้องแยกตัวเพื่อสังเกตอาการอย่างน้อย 14 วัน				Kn11 ( )
12. การกักตัวผู้ติดเชื้อไวรัสโคโรนา 2019 เป็นวิธีการลดการแพร่กระจายเชื้อไวรัสที่มีประสิทธิภาพ				Kn12 ( )

BUU-IRB Approved
21 May 2021

ส่วนที่ 4 แบบสัมภาษณ์การรับรู้ความเชื่อด้านสุขภาพเกี่ยวกับโรคติดเชื้อไวรัสโคโรนา 2019

คำชี้แจง ผู้สัมภาษณ์ทำการสัมภาษณ์ผู้สูงอายุตามข้อคำถามเกี่ยวกับ**การรับรู้ความเชื่อด้านสุขภาพเกี่ยวกับโรคติดเชื้อไวรัสโคโรนา 2019** แล้วให้ทำเครื่องหมาย ✔ ในข้อที่ตรงกับความเชื่อด้านสุขภาพที่ผู้สูงอายุระบุมาดังนี้

เห็นด้วยอย่างยิ่ง หมายถึง ผู้สูงอายุมีความรู้สึกตรงกับข้อความเป็นอย่างมาก
เห็นด้วย หมายถึง ผู้สูงอายุมีความรู้สึกตรงกับข้อความนั้น
ไม่แน่ใจ หมายถึง ผู้สูงอายุมีความรู้สึกไม่แน่ใจกับข้อความนั้น
ไม่เห็นด้วย หมายถึง ผู้สูงอายุมีความรู้สึกไม่เห็นด้วยกับข้อความนั้น

**การรับรู้ความเชื่อด้านสุขภาพ**	เห็นด้วยอย่างยิ่ง	เห็นด้วย	ไม่แน่ใจ	ไม่เห็นด้วย
**การรับรู้โอกาสเสี่ยงของการเป็นโรค**				
1. ท่านไม่ได้ให้ความสนใจกับโรคติดเชื้อไวรัสโคโรนา 2019 และยังคงใช้ชีวิตตามปกติ (-)				
2. ท่านคิดว่าท่านมีโอกาสจะติดเชื้อไวรัสโคโรนา 2019 ได้ง่ายกว่าคนอื่น (+)				
3. ท่านมีความวิตกกังวลว่าตนเองมีความเสี่ยงในการติดเชื้อไวรัสโคโรนา 2019 (+)				
4. สุขภาพของท่านในปัจจุบันทำให้ท่านมีโอกาสในการติดเชื้อไวรัสโคโรนา 2019 ได้ง่าย				
5. ท่านคิดว่าเป็นไปไม่ได้ที่ท่านจะติดเชื้อไวรัสโคโรนา 2019				
**การรับรู้ความรุนแรงของโรค**				
6. ท่านกลัวติดเชื้อไวรัสโคโรนา 2019 เพราะทำให้มีโอกาสการเสียชีวิตสูงมาก				
7. ท่านกลัวติดเชื้อไวรัสโคโรนา 2019 จนทำให้ท่านวิตกกังวลและนอนไม่หลับ				
8. ท่านกลัวติดเชื้อไวรัสโคโรนา 2019 เพราะเป็นโรคที่สามารถแพร่กระจายและทำให้คนติดเชื้อได้ง่าย				
9. ท่านกลัวติดเชื้อไวรัสโคโรนา 2019 เพราะเป็นโรคที่ยังไม่มียาที่รักษาให้หายหรือวัคซีนที่ป้องกันโรคที่มีประสิทธิภาพ				
10. การเชื้อไวรัสโคโรนา 2019 จะทำให้ท่านต้องกักตัว หรือ ถูกแยกตัวออกจากสังคม				

Version 2.0/ May 20, 2021

BUU-IRB Approved
21 May 2021

การรับรู้ความเชื่อด้านสุขภาพ	เห็นด้วยอย่างยิ่ง	เห็นด้วย	ไม่แน่ใจ	ไม่เห็นด้วย
**การรับรู้ถึงประโยชน์ของการป้องกันโรค**				
11. การสวมใส่อุปกรณ์ป้องกันตนเองเช่น หน้ากากอนามัย ทั้งแบบผ้า และแบบใช้แล้วทิ้ง หรือ ใส่ถุงมือที่ใช้แล้วทิ้ง เป็นการป้องกันการติดเชื้อไวรัสโคโรนา 2019				
12. การล้างมือบ่อย ๆ ด้วยน้ำและสบู่ สามารถป้องกันการติดเชื้อไวรัสโคโรนา 2019 ได้				
13. การหลีกเลี่ยงในการไปสถานที่ที่มีคนจำนวนมาก ๆ เช่น สวนสาธารณะ ตลาดสด ห้างสรรพสินค้า หรือ วัด สามารถป้องกันการติดเชื้อไวรัสโคโรนา 2019 ได้				
14. การเว้นระยะห่างจากผู้อื่น 1-2 เมตร สามารถป้องกันการติดเชื้อไวรัสโคโรนา 2019 ได้				
15. การอาบน้ำ สระผม เปลี่ยนเสื้อผ้าทันทีเมื่อกลับมาจากการทำธุระนอกบ้านช่วยลดโอกาสการได้รับเชื้อไวรัสโคโรนา 2019				
**การรับรู้อุปสรรคในการป้องกันโรค**				
16. ในการปฏิบัติตนเพื่อป้องกันตนเองจากการติดเชื้อไวรัสโคโรนา 2019 ในการดำเนินชีวิตประจำวัน เป็นเรื่องที่ยุ่งยากมากสำหรับท่าน				
17. การล้างมือด้วยสบู่ หรือเจลล้างมือ ทุกครั้งหลังสัมผัสสิ่งของสาธารณะ เป็นเรื่องยุ่งยากและไม่สะดวกสำหรับท่าน				
18. การสวมหน้ากากอนามัยเมื่อออกไปนอกบ้านทุกครั้งเป็นเรื่องยุ่งยากและไม่สะดวกสำหรับท่าน				
19. การไม่ใช้มือสัมผัสหน้า จมูกและปาก เป็นเรื่องที่ยุ่งยากสำหรับท่าน				
20. ระหว่างที่มีการระบาดของเชื้อไวรัสโคโรนา 2019 การออกไปซื้อของที่ตลาด หรือ ร้านค้า หรือห้างสรรพสินค้า ทำให้ท่านเกิดความยุ่งยาก				
**การรับรู้ข้อมูลข่าวสารเกี่ยวกับโรคติดเชื้อไวรัสโคโรนา 2019 (ปัจจัยชักนำการปฏิบัติ)**				
21. การรับรู้ข้อมูลข่าวสารจากโทรทัศน์ วิทยุ เอกสารแผ่นพับ หนังสือพิมพ์ วารสารต่าง ๆ สื่อทางอิเล็กทรอนิกส์(Facebook) และกลุ่มโซเชียลมีเดีย (Line) ทำให้ท่านมีความรู้ มีความเข้าใจถึงวิธีการป้องกันตนเองจากการติดเชื้อไวรัสโคโรนา 2019				
22. เจ้าหน้าที่หรือบุคลากรด้านสาธารณสุข ให้คำแนะนำหรือให้การปรึกษาการปฏิบัติตัวในการป้องกันการติดเชื้อไวรัสโคโรนา 2019				
23. สมาชิกในครอบครัวของท่านให้คำแนะนำหรือให้การปรึกษาการปฏิบัติตัว ในการป้องกันการติดเชื้อไวรัสโคโรนา 2019				

BUU-IRB Approved
21 May 2021
