# Supplementary material for: Preventive behaviors of COVID-19 during the COVID-19 pandemic among community-dwelling older adults in Thailand
Source: PeerJ. 2025 May 13;13:e19412. doi: 10.7717/peerj.19412 (PMC12083464; doi:10.7717/peerj.19412)
Supplement: Supplemental Information 3 [file peerj-13-19412-s003.docx]

# COVID-19 Prevention Behavior Questionnaire

## Research Title:

## Preventive Behaviors of COVID-19 during the COVID-19 Pandemic Among Community-Dwelling Older Adults in Thailand

## Instructions:

This questionnaire is divided into 4 sections:
1. Personal Information
2. COVID-19 Prevention Behavior Interview
3. Knowledge About COVID-19 Interview
4. Perceptions and Beliefs About COVID-19 Health Impacts Interview

The interviewer will record the responses or check √ in the appropriate box ( ) and fill in any required blanks. All data collected is confidential and will not harm the respondent. Researchers will analyze the data and present it in an aggregated form for the study.

Date: 21 May 2021

## Section 1: Personal Information

1. Gender

( ) Male
( ) Female

2. Current Age: ___ years

3. Marital Status

( ) Married
( ) Single
( ) Widowed
( ) Divorced/Separated

4. Religion

( ) Buddhist
( ) Muslim
( ) Christian
( ) Other: __________

5. Occupation

( ) Unemployed
( ) Housewife
( ) Agriculture
( ) Laborer
( ) Business/Trade
( ) Retired Government Official
( ) Other: __________

6. Average Monthly Income: ___ Baht

7. Education Level

( ) None
( ) Primary (Grade 4)
( ) Primary (Grade 6)
( ) Secondary (Junior High)
( ) Secondary (Senior High/Vocational)
( ) Diploma/Vocational Certificate
( ) Bachelor's Degree
( ) Postgraduate
( ) Other: __________

8. How would you describe your current health status?

( ) Excellent
( ) Good
( ) Fair
( ) Poor

9. Do you have any chronic illnesses? If yes, specify:

( ) None
( ) Hypertension
( ) Diabetes
( ) Cardiovascular Disease
( ) Chronic Obstructive Pulmonary Disease (COPD)
( ) High Cholesterol
( ) Gout
( ) Allergies
( ) Joint/Bone Pain
( ) Chronic Kidney Disease
( ) Other: __________

## Section 2: COVID-19 Prevention Behavior Interview

Instructions: The interviewer should ask the elderly respondents about their COVID-19 prevention behaviors. Mark the box (α) corresponding to their responses based on the following scoring system:
- Consistently practiced: The respondent practices the activity every time. (3 points)
- Frequently practiced: The respondent practices the activity most of the time. (2 points)
- Occasionally practiced: The respondent practices the activity sometimes. (1 point)
- Never practiced: The respondent has never practiced the activity. (0 points)

### COVID-19 Prevention Behaviors

| COVID-19 Prevention Behaviors | Consistently Practiced (3 points) | Frequently Practiced (2 points) | Occasionally Practiced (1 point) | Never Practiced (0 points) |
| --- | --- | --- | --- | --- |
| 1. Do you always wear a face mask or cloth mask when leaving the house for errands? (BH1) |  |  |  |  |
| 2. Do you wash your hands with soap or use alcohol-based hand sanitizer after touching shared items, before eating, or after using the restroom? (BH2) |  |  |  |  |
| 3. Do you avoid touching your face, eyes, mouth, and nose with your hands? (BH3) |  |  |  |  |
| 4. During the pandemic, do you avoid crowded places such as markets, temples, shopping malls, or community events? (BH4) |  |  |  |  |
| 5. Do you exercise at least 3 days a week to maintain good health and prevent COVID-19? (BH5) |  |  |  |  |
| 6. Do you eat a balanced diet with five food groups in at least 3 meals daily to stay healthy and prevent COVID-19? (BH6) |  |  |  |  |
| 7. Do you consume traditional herbal remedies, either Thai or Chinese, to prevent COVID-19? (BH7) |  |  |  |  |
| 8. Do you take vitamins, supplements, or antibiotics to prevent COVID-19? (BH8) |  |  |  |  |

## Section 3: Knowledge About COVID-19

Instructions: The interviewer should ask the elderly respondents questions about their knowledge of COVID-19. Mark the box (α) corresponding to their responses:
- Yes: The statement matches the respondent’s understanding.
- No: The statement does not match the respondent’s understanding.
- Not sure: The respondent is unsure or does not know the answer.

### Knowledge Statements

| Knowledge Statements | Yes | No | Not Sure |
| --- | --- | --- | --- |
| 1. Regular use of face masks and frequent hand washing can help prevent the spread of COVID-19. (Kn1) |  |  |  |
| 2. When an infected person coughs or sneezes in public, it can easily transmit COVID-19 to others nearby. (Kn2) |  |  |  |
| 3. Having immunity against COVID-19 can protect individuals from infection. (Kn3) |  |  |  |
| 4. Avoiding crowded places or large gatherings helps reduce the risk of contracting COVID-19. (Kn4) |  |  |  |
| 5. Symptoms of COVID-19 include fever, fatigue, dry cough, and muscle aches. (Kn5) |  |  |  |
| 6. COVID-19 causes less nasal congestion and runny nose compared to the common cold. (Kn6) |  |  |  |
| 7. Currently, there is no effective treatment for COVID-19, except self-protection against exposure. (Kn7) |  |  |  |
| 8. Elderly individuals with chronic illnesses or obesity are at higher risk of severe symptoms if infected with COVID-19. (Kn8) |  |  |  |
| 9. COVID-19 spreads through droplets released by infected individuals when coughing or sneezing. (Kn9) |  |  |  |
| 10. Asymptomatic individuals with COVID-19 cannot transmit the virus to others. (Kn10) |  |  |  |
| 11. Individuals in contact with COVID-19 patients should isolate themselves for at least 14 days to monitor symptoms. (Kn11) |  |  |  |
| 12. Isolating infected individuals effectively reduces the spread of COVID-19. (Kn12) |  |  |  |

## Section 4: Perceptions About COVID-19 Health Beliefs

Instructions: The interviewer should ask the elderly respondents about their perceptions regarding health beliefs and COVID-19. Mark the box (α) corresponding to their responses:
- Strongly agree: The respondent strongly feels the statement is accurate.
- Agree: The respondent feels the statement is accurate.
- Not sure: The respondent is unsure about the statement.
- Disagree: The respondent does not feel the statement is accurate.

### Health Belief Statements

| Perceived susceptibility | | | | |
| --- | --- | --- | --- | --- |
| Statement | Strongly agree | Partially agree | Partially disagree | Strongly disagree |
| Perceived susceptibility | | | | |
| 1. I’m not concerned about COVID-19 and do my daily activities like before. |  |  |  |  |
| 2. I am more likely to get an infection. |  |  |  |  |
| 3. I consider myself to be at risk of COVID-19. |  |  |  |  |
| 4. My health may be at risk if I get the disease. |  |  |  |  |
| 5. I think it is impossible for me to get the disease. |  |  |  |  |
| Perceived severity | | | | |
| 1. COVID-19 has a high death rate. It scares me. |  |  |  |  |
| 2. I am worried about the disease and cannot sleep well. |  |  |  |  |
| 3. I am worried about getting the disease because it can easily spread. |  |  |  |  |
| 4. I am worried about getting the disease because there is no effective vaccine or medicine. |  |  |  |  |
| 5. If I get the infection, I have to isolate from society. |  |  |  |  |
| Perceived benefits | | | | |
| 1. COVID-19 can be prevented easily with personal protective equipment, such as masks and disposable gloves. |  |  |  |  |
| 2. COVID-19 can be prevented easily by washing hands regularly with soap and water. |  |  |  |  |
| 3. COVID-19 can be prevented by avoiding meeting people in crowded places. |  |  |  |  |
| 4. COVID-19 can be prevented easily by keeping social distancing. |  |  |  |  |
| 5. COVID-19 can be prevented easily by taking a shower or bath. |  |  |  |  |
| Perceived barriers | | | | |
| 1. It is difficult to follow the instructions to prevent this disease. |  |  |  |  |
| 2. It is difficult to wash hands regularly with soap and water. |  |  |  |  |
| 3. To wear a surgical mask or cloth mask outside the household is difficult. |  |  |  |  |
| 4. It is easy to touch my mouth, nose, and eyes. |  |  |  |  |
| 5. Shopping at the market and having to prevent the disease are difficult. |  |  |  |  |
| Cues to action | | | | |
| 1. TV, Facebook, Line, and radio information about COVID-19 has been helpful. |  |  |  |  |
| 2. The recommendation to prevent the disease from healthcare workers has been helpful. |  |  |  |  |
| 3. The recommendation to prevent the disease from household members has been helpful. |  |  |  |  |
